# Exploring Metabolite Changes in Crispy Tilapia During the Crisping Process via ^1^H-NMR Metabolomic Analysis

**DOI:** 10.3390/foods15071232

**Published:** 2026-04-04

**Authors:** Fanshu Cheng, Ling Zhang, Xueyan Li, Manni Zheng, Xiaoyan Xu, Xingguo Tian

**Affiliations:** 1 Guangdong Provincial Key Laboratory of Food Quality and Safety, Nation-Local Joint Engineering Research Center for Machining and Safety of Livestock and Poultry Products, South China Agricultural University, Guangzhou 510642, China; fanshucheng2026@outlook.com (F.C.); nomino2026@163.com (M.Z.); 2 College of Biological and Food Engineering, Guangdong University of Petrochemical Technology, Maoming 525000, China; mmzhl1130@126.com; 3 Zhongshan Institute, College of Materials and Food, University of Electronic Science and Technology of China, Zhongshan 528402, China; xueyan9494@163.com

**Keywords:** crispy tilapia, faba beans, biomarkers, ^1^H-NMR, multivariate analysis, type I collagen

## Abstract

Faba bean-fed crispy tilapia represents a commercially valuable aquaculture product, renowned for its exceptional muscle firmness. However, the dynamic changes in muscle metabolite profiles during the tilapia crisping process remain largely unelucidated. In this study, proton nuclear magnetic resonance spectroscopy (^1^H-NMR) combined with multivariate statistical analysis was employed to characterize and compare the muscle metabolomes of tilapia subjected to different crispness grades (CD0, CD2, CD4). A total of 11 differential metabolites were successfully identified, among which glycine, threonine, and trans-4-hydroxy-L-proline were demonstrated to be potential crispness-related biomarkers. Specifically, as the crispness grade increased from 0 to 4, the muscle contents of these key metabolites exhibited a consistent downward trend: glycine decreased significantly from 19.86 mM to 7.15 mM, threonine from 1.21 mM to 0.58 mM, and trans-4-hydroxy-L-proline from 2.25 mM to 0.89 mM. Subsequent metabolic pathway enrichment analysis further revealed that the glycine-serine-threonine metabolic pathway represented the most significantly perturbed pathway associated with the crisping process. Collectively, our findings demonstrate that faba bean-based feeding regimens enhance tilapia muscle crispness by orchestrating metabolite signatures involved in collagen biosynthesis and lipid metabolism. These results not only provide novel insights into the intrinsic molecular mechanisms underlying tilapia crisping but also establish a solid theoretical framework for the precise quality control and standardized production of high-quality crispy tilapia.

## 1. Introduction

Tilapia (*Oreochromis mossambicus*), renowned for its superior nutritional value characterized by high protein content, strong reproductive capacity, rapid growth, and exceptional environmental resilience, has emerged as one of the most economically important finfish species in global commercial aquaculture, particularly in developing countries [1]. As the world’s largest tilapia exporter, China has significantly expanded its global market share through intensive aquaculture practices. However, the continuous elevation of culture stocking density has exacerbated intraspecific competition for food and spatial resources, which consequently exerts adverse impacts on the feeding behaviour, growth performance, physiological status, metabolic processes, and immune function of cultured fish [2]. Such issues have ultimately resulted in persistently inferior muscle quality and drastically depressed market values of tilapia products [3]. Therefore, the development and production of high-quality and high-value tilapia products are of paramount significance for the sustainable development of the global tilapia aquaculture industry.

In recent years, increasing research attention has been devoted to improving fish muscle quality via faba bean-based feeding regimens. Meat texture is an important organoleptic indicator of meat quality, as it exerts a significant influence on consumers’ overall eating experience and satisfaction [4]. Texture Profile Analysis (TPA) simulates oral mastication movements and enables the objective evaluation of meat texture through quantitative indicators [5]. Studies have shown that feeding tilapia with faba beans can significantly enhance the physicochemical properties and nutritional value of the fish’s muscle: it increases the hardness, chewiness, gumminess, and fatty acid content of Nile tilapia, while significantly reducing the crude fat content [6]. Furthermore, tilapia fed with faba beans exhibit reduced oxidative damage to hepatic proteins and intestinal tissues, thereby improving their overall physiological health [7]. Compared with ordinary tilapia, crispy tilapia offers distinct advantages, including a short culture cycle, intact texture (not mushy) after cooking, and a crispy taste. These characteristics have boosted market demand for crispy tilapia and substantially improved its economic benefits [8]. Faba bean seeds are rich in protein (20.3–41%), starch, and essential minerals, making them a potential high-quality protein and energy source for aquafeeds [9]. The incorporation of faba beans into formulations helps reduce feed costs while ensuring a stable feed supply, particularly as a viable substitute for soybean meal in aquatic diets [10]. In addition, the market price of crispy tilapia is more than threefold higher than that of ordinary tilapia.

Crispy tilapia farming exhibits enormous potential in China but remains in its nascent stage. Insufficient crisping duration results in suboptimal flesh crispness, yet prolonging the crisping period leads to a significant reduction in fish survival rates. Therefore, it is of great significance to elucidate the pathways through which faba beans modulate fish quality and identify the key substances influencing crispness changes. This knowledge will facilitate the optimization of aquafeed formulations and enable effective quality monitoring of high-quality farmed tilapia. However, most existing studies have focused on investigating the effects of different faba bean supplementation levels on tilapia’s growth performance, muscle quality, serum biochemical parameters, and oxidative/immune profiles [11,12,13]. Although studies have demonstrated that different faba bean meal supplementation regimens can alter muscle quality and expression of collagen-related genes in grass carp [14,15], the key molecules involved and the interactive mechanisms by which these molecules regulate crispness changes remain largely unclear. In addition, empirical studies indicate that approximately 20% of globally traded seafood products may be subject to fraudulent practices, including species substitution, mislabeling of geographic origin, and misrepresentation of production methods [16,17]. As crispy tilapia commands a significant price premium, it may become a prime target for economic adulteration. The identification of species-specific or process-specific biomarkers through metabolomic approaches could facilitate the development of authentication tools for value-added aquaculture products [18,19].

Metabolomics enables the generation of comprehensive metabolic profiles of food products and facilitates the analysis of how different conditions impact food quality. The quantification of differential metabolites and subsequent pathway analysis allows for the monitoring of quality changes that are difficult to measure with a single indicator. This provides critical guidance for investigating the crispification mechanism and evaluating the crispness of crispy tilapia. Compared with other analytical techniques, NMR has garnered increasing attention due to its distinct advantages, including simple sample pretreatment, minimal sample damage and consumption, stable and rapid detection, and the feasibility of real-time dynamic monitoring [20]. In recent years, NMR-based metabolomics, particularly ^1^H NMR spectroscopy, has played an increasingly prominent role in characterizing complex food matrices. Accordingly, ^1^H NMR has been successfully utilized for the evaluation and control of aquatic product quality [21,22,23,24]. The application of ^1^H NMR enables the simultaneous detection and quantification of metabolites with diverse chemical properties, thereby eliminating the need for multiple sample treatments and ensuring data reliability and stability. To the best of our knowledge, NMR-based metabolomics has not yet been applied to characterize the metabolite profiles of crispy tilapia at different crispness stages.

In this study, ^1^H-NMR spectroscopy combined with multivariate statistical analysis was employed to identify and compare the muscle metabolite profiles of crispy tilapia with different crispness grades, as well as with those of ordinary tilapia, and to explore potential crispness-associated biomarkers. The insights obtained from metabolite profiling will inform the development of crispness-enhancing aquafeeds and the establishment of reliable crispness assessment methods, thereby promoting the sustainable production of high-quality, high-yield crispy tilapia.

## 2. Materials and Methods

### 2.1. Samples Obtainment and Preparation

Two tilapia varieties, namely conventional and crispy strains, were acquired from Guangdong Youpei Supply Chain Management Co., Ltd. (Zhongshan, China). Fresh tilapia fillets were transported to the laboratory under refrigerated conditions. The fillets were derived from commercial aquaculture production, and no animal experimentation or ethical approval was required for this study. All experimental fish were reared under consistent environmental and management conditions to eliminate confounding variables. Crispy tilapia were fed a specialized crisping diet purchased from Jingzhou Harvest Agricultural Science and Technology Co., Ltd. (Jingzhou, China), which contained approximately 60% faba beans as the key component. In contrast, ordinary tilapia were supplied with a commercial compound feed from Guangzhou Hope Feed Co., Ltd. (Guangzhou, China), with no faba bean inclusion. Detailed formulations of the two experimental diets are presented in Table S1. The daily feeding ration was 1.5–2% of the total fish biomass in the pond, divided equally into three meals per day. The feeding amount was dynamically adjusted based on ambient weather conditions and the gastrointestinal health status of the fish.

Sensory evaluation was performed by qualified professionals following the established criteria shown in Table S2. Sensory crispness data were obtained from the internal quality control records of Guangdong Youpei Supply Chain Management Co., Ltd. Assessments were conducted by trained panellists as part of routine product quality testing. All data were anonymized before being accessed by the authors, and no personal information of the panellists was collected or utilized. No direct contact with human subjects was involved in this study. Sensory crispness data were used exclusively for sample grouping purposes. Fish fillets graded as grade 0 (CD0), grade 2 (CD2), and grade 4 (CD4) were selected as experimental samples. A total of 48 fillets were analyzed, with 16 biological replicates per grade. Fish in the CD0 group (mean weight = 250 ± 15.25 g) were fed a conventional diet for approximately 30 days; the CD2 group (mean weight = 450 ± 16.44 g) was fed for around 60 days, including 30 days of the crisping diet; and the CD4 group (mean weight = 1000 ± 55.63 g) was fed for roughly 120 days, including 90 days of the crisping diet. Among the three groups, the CD4 group represents commercially available standard products. After slaughter, the fish were processed following a standardized protocol: the heads, bones, and viscera were removed, and only the white dorsal skeletal muscle was retained for subsequent analysis. A 5 g sample of white skeletal muscle was collected from the dorsal portion of each fillet. Each sample was individually homogenized, lyophilized for 48 h, and then stored at −80 °C.

### 2.2. Chemicals

2,2,3,3-Tetramethylsilane propionate (TSP) and deuterium oxide (D_2_O) were purchased from Maclean Reagents (Beijing, China), while hematoxylin-eosin (H&E) staining kit and Sirius Red staining kit were obtained from Solebro Technology (Beijing, China). All chemicals and solvents used for sample treatment and analysis were of analytical grade.

### 2.3. Texture Profile Analysis (TPA)

TPA was performed on the three tilapia groups using a Texture Analyzer (Surface Measurement Systems, EZ TEST, London, UK). Briefly, fish fillets were cut into uniform cubes of 2.0 cm × 2.0 cm × 1.5 cm in size. The following parameters were set to determine hardness, springiness, cohesiveness, chewiness, resilience, and gumminess: downward compression speed of 2 mm/s, compression speed of 10 mm/s, return speed of 2 mm/s, probe trigger force of 0.05 N, and compression degree of 30%. For each crispness grade, 14 parallel experiments were conducted, including 7 for raw samples and 7 for cooked samples. Raw and cooked samples in each group were taken from the same individual fillet. Cooked fillets were prepared by steaming in a covered boiling water bath for 5 min, followed by natural cooling at room temperature for 15 min [25].

### 2.4. Comprehensive Evaluation of Crispness Based on the Entropy Weight Method

The entropy weight method (EWM) was used to calculate the weights of the TPA indicators based on their interrelationships. The crispness grades determined by sensory evaluation were evaluated using the composite index (CI). The specific calculation procedures and formulas are presented in Appendix A.

### 2.5. Histological Analysis

Samples of white dorsal skeletal muscle were collected perpendicular to the long axis of the muscle fibres, and the tissues were fixed in 4% paraformaldehyde for subsequent analysis. Following fixation, the tissues were embedded in paraffin and sectioned at a thickness of 4 µm for subsequent staining assays. Hematoxylin and Eosin (H&E) staining combined with Sirius Red staining was used to distinguish between muscle fibres and collagen. Observations were carried out using a light microscope (Leica, Wetzlar, Germany). Collagen types I and III were discriminated and quantified under polarized light. Relative quantification of type I and type III collagen was performed using Image Pro Plus 6.0 software. In hue-saturation-intensity (HSI) mode, the red and green regions were selected by adjusting the hue (H) value. Once the red or green regions were accurately selected, the corresponding area was measured, and the percentage of each collagen type relative to the total tissue area was calculated. For each crispness grade, three individual fish fillets were analyzed for both H&E staining and Sirius Red staining.

### 2.6. ^1^H NMR Analysis

#### 2.6.1. Sample Preparation for ^1^H NMR

^1^H NMR analysis was performed with slight modifications based on the methods described by Shen [26] and Zhao [27]. Briefly, 150 mg of lyophilized tilapia muscle samples were homogenized with 1.1 mL of ultrapure water and 1.14 mL of methanol for 2 min, followed by sonication in an ice bath for 7 min to extract metabolites. The resulting mixture was centrifuged at 10,000× *g* for 10 min at 4 °C (GL–23M, Xiangyi, Hunan, China). The supernatant was collected and lyophilized. The lyophilized powder was reconstituted in 800 μL of phosphate buffer (90 mM, pH 7.4) prepared with 100% deuterium oxide (D_2_O), which contained 0.02% (w/v) 2,2,3,3-tetramethylsilane propionate (TSP). After centrifugation at 10,000× *g* for 10 min at 4 °C, the supernatant was transferred to a 5 mm NMR tube for NMR analysis. TSP was used as an internal chemical shift reference. All procedures were performed on ice to prevent metabolite degradation.

#### 2.6.2. NMR Experiments

Fish muscle metabolites were analyzed using a NMR spectrometer equipped with a cryoprobe (Avance NEO 600, Bruker Biospin AG, Fällanden, Switzerland), operating at 600.13 MHz. The standard presaturation NOESY pulse sequence (noesygppr1d) from the Bruker library was employed for water suppression. The acquisition parameters were as follows: The acquisition parameters were as follows: spectral width = 12 kHz, number of sampling points = 32 K, acquisition time = 2.8 s, relaxation delay = 5 s, mixing time = 10 ms, and number of scans = 64. Spectral processing was carried out using MestReNova (Version 14.0, Mestrelab Research, Santiago de Compostela, Spain). All free induction decays (FIDs) were zero-filled to 64 K data points, and the signal was exponentially windowed with a linewidth factor of 0.3 Hz. The spectra were then subjected to phase and baseline correction.

#### 2.6.3. ^1^H NMR Data Processing for Statistical Analysis

^1^H NMR spectra were processed using MestReNova. The chemical shift in the internal standard TSP peak was calibrated to 0.00 ppm. The residual water peak (4.7–5.1 ppm), residual methanol peak (3.34–3.37 ppm), TSP peak (0.00 ppm), and peak-free baseline regions were manually removed. The spectra were binned at an interval of 0.02 ppm and normalized to the total area. The integrated equal-width bins were imported into Microsoft Excel as a data matrix for subsequent statistical analysis.

#### 2.6.4. Multivariate Statistical Analysis

The NMR data were imported into SIMCA-P software (Version 14.1, Umetrics, Umeå, Sweden) for multivariate statistical analysis, including principal component analysis (PCA) and orthogonal partial least squares-discriminant analysis (OPLS-DA). Variables were scaled to “Unit Variance” (i.e., the weighting factor was calculated as 1/sdj, where 1/sdj denotes the standard deviation of the j-th variable around its mean value). The results were visualized as two-dimensional (2D) score scatter plots, with each point representing an individual sample. OPLS-DA was subsequently employed to delineate metabolic differences between groups. Model validation was performed using a permutation test (200 permutations).

#### 2.6.5. Metabolite Identification and Quantification

Metabolites were identified using the standard compound peak database in Chenomx NMR Suite (Version 9.02, Chenomx Inc., Edmonton, AB, Canada). Tentative assignments were further verified by cross-referencing with the Human Metabolome database (HMDB) (https://hmdb.ca) and PubChem database (https://pubchem.ncbi.nlm.nih.gov), with a focus on metabolites previously reported in fish muscle tissue. For unidentified signals, characteristic functional group information was obtained from HMDB and PubChem to support structural interpretation. Raw spectra were used for the relative quantification of differential metabolites, with TSP (1.175 mM) as the internal reference. This approach is based on the principle that the signal area is proportional to the number of protons in the sample.

#### 2.6.6. Putative Identification and Analysis of Differential Metabolites

Differential metabolites with high potential for discriminating crispness were screened via OPLS-DA, using the selection criteria of variable importance in projection (VIP) score > 1.00, fold change (FC) > 2.00, and *p*-value < 0.05. These VIP variables corresponded to spectral regions (integrated bins) with a width of 0.02 ppm. Subsequently, a Venn diagram was constructed to assess the overlap of differential metabolites among tilapia samples with different crispness grades. Finally, the corresponding spectral regions were matched to the previously identified tilapia metabolites using Chenomx NMR Suite.

Pathway analysis and enrichment analysis of differential metabolites were conducted using the MetaboAnalyst 5.0 online data analysis platform (https://www.metaboanalyst.ca/), based on the Kyoto Encyclopedia of Genes and Genomes (KEGG) database.

### 2.7. Statistical Analysis

All experiments were performed with at least three independent biological replicates, and the data were analyzed using one-way analysis of variance (ANOVA). Subsequently, Duncan’s multiple range test was used for multiple comparisons to determine significant differences among groups, with a *p*-value < 0.05 considered statistically significant. The results were expressed as the mean ± standard error of the mean (mean ± SEM). Unless otherwise specified, the relevant graphs were generated using Origin 2024, and statistical analyses were conducted using IBM SPSS Statistics 26.0 (Armonk, NY, USA).

## 3. Results

### 3.1. Texture Profile Analysis (TPA)

The TPA results of crispy tilapia with different crispness grades are presented in Table 1. TPA revealed significant differences in texture attributes, including hardness, cohesiveness, and chewiness, among tilapia groups with different crispness grades. Significantly higher values (*p* < 0.05) for these texture attributes were observed in the CD4 group (high crispness) compared with the CD0 group (ordinary crispness). The texture properties of raw and cooked fish fillets exhibited substantial changes: the hardness, gumminess, and chewiness of raw fillets increased by 1.1-, 1.2-, and 0.8-fold, respectively, while those of cooked fillets increased by 1.5-, 2.8-, and 3.4-fold, respectively. The enhancement in chewiness was particularly pronounced in cooked fillets. However, no significant differences in chewiness or gumminess of cooked fillets were observed between the 30-day (CD2) and 90-day (CD4) faba bean-fed groups.

**Figure 1:**
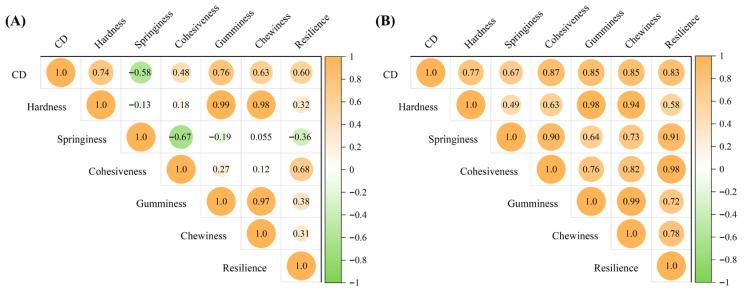
Correlation heatmap showing the relationships between texture profile analysis (TPA) indices and crispness grades of raw (**A**) and cooked (**B**) tilapia fillets.

Figure 1 illustrates the correlation between Texture Profile Analysis (TPA) indicators and the crispness grade of raw and cooked tilapia fillets. For raw fillets, the crispness of tilapia showed a strong positive correlation with hardness and chewiness, and a negative correlation with springiness. For cooked fillets, the crispness of crispy tilapia exhibited a strong positive correlation with all TPA indicators. Among these indicators, the correlation coefficients of cohesiveness, chewiness, gumminess, and resilience exceeded 0.8.

The results of weighting the TPA indicators of cooked tilapia fillets using the entropy weight method (EWM) are presented in Table 2. Among these TPA indicators, hardness, chewiness, and gumminess were assigned the highest weights, with values of 22.96%, 21.72%, and 20.49%, respectively. Ultimately, the crispness can be expressed by the composite index (CI), calculated as follows: CI = Hardness × 0.2296 + Springiness × 0.0754 + Cohesiveness × 0.1326 + Gumminess × 0.2049 + Chewiness × 0.2172 + Resilience × 0.1403.

**Table 2:** Entropy Weight Method (EWM) weights assigned to texture profile analysis (TPA) Indicators for Cooked Tilapia Fillets.

TPA Index	Entropy Weight
Hardness	22.96%
Springiness	7.54%
Cohesiveness	13.26%
Gumminess	20.49%
Chewiness	21.72%
Resilience	14.03%

### 3.2. Characterization of Muscle Tissue Metabolome

**Figure 2:**
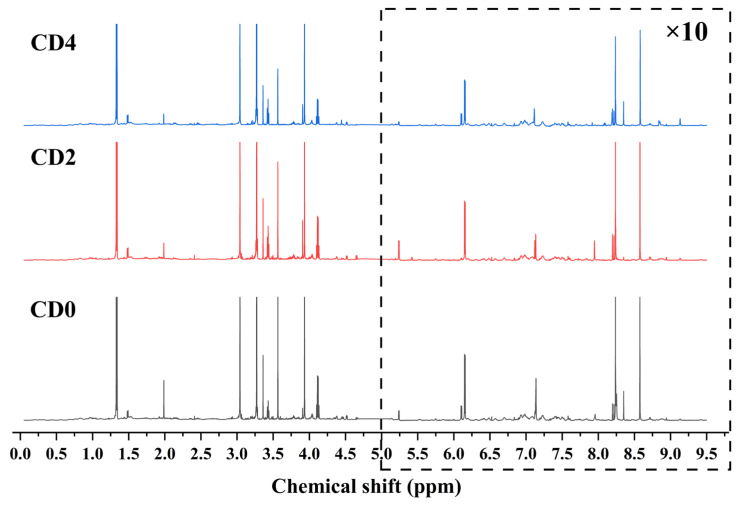
^1^H-NMR typical spectra of tilapia with different crispness grades (CD0, CD2, and CD4).

This study analyzed small-molecule metabolites in crispy tilapia muscle tissue across three groups (CD0, CD2, and CD4). The ^1^H NMR spectra of the samples were acquired, and each metabolite was identified. Visual inspection of the spectra revealed distinct differences in the representative profiles among crispy tilapia with different crispness grades (Figure 2). A total of 51 metabolites were identified, primarily including amino acids, peptides, organic acids, glycans, nucleosides, nucleotides, and their derivatives (Table 3).

### 3.3. Characterization of Muscle Tissue Metabolome

**Figure 3:**
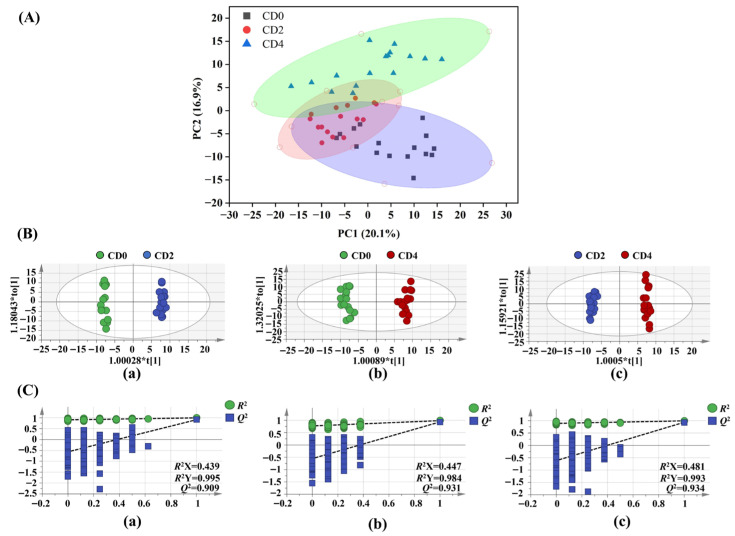
Multivariate statistical analysis of ^1^H-NMR metabolomic data from tilapia muscle tissue with different crispness grades (CD0, CD2, and CD4, *n* = 16 per group). (**A**) Principal component analysis (PCA) score plot; (**B**) Orthogonal partial least squares discriminant analysis (OPLS-DA) score plot; (**C**) Permutation test (200 permutations) validating the OPLS-DA model. Note: Each dot represents a fish fillet.

The PCA score plot derived from ^1^H NMR data (Figure 3A) exhibited a discernible trend in classifying tilapia groups according to varying crispness grades. PCA, an unsupervised multivariate statistical method, was used to visualize data distribution without presupposing group characteristics. The relevant statistical parameters are as follows: *R^2^X* = 0.621, *R^2^Y* = 0.365. The first two principal components (PCs) explained 20.1% and 16.9% of the total variance, respectively. All sample points fell within the 95% confidence interval, indicating no outlier samples. The CD0 and CD4 groups were clearly distinguished from each other; however, there was still some overlap among the three groups.

Subsequently, to maximize the variation between groups, a supervised OPLS-DA method was used to conduct detailed pairwise group comparisons. This method enhanced the separation of features between groups and assessed differences in metabolite profiles among muscle extracts of crispy tilapia with different crispness grades. High *R^2^* and *Q^2^* values indicate significant differences between paired groups and good overall predictability of the fitted models. The score plot in Figure 3B clearly demonstrates that the CD0, CD2, and CD4 groups are distinguishable from one another, suggesting substantial differences in metabolite compositions among the three groups. The reliability of the model was validated through a permutation test (Figure 3C). The results showed high model fitting accuracy, clear separation between groups, and distinct intra-group clustering. No overfitting was detected, indicating the model’s robust predictive capability and reliability.

### 3.4. Screening for Differential Biomarkers

**Figure 4:**
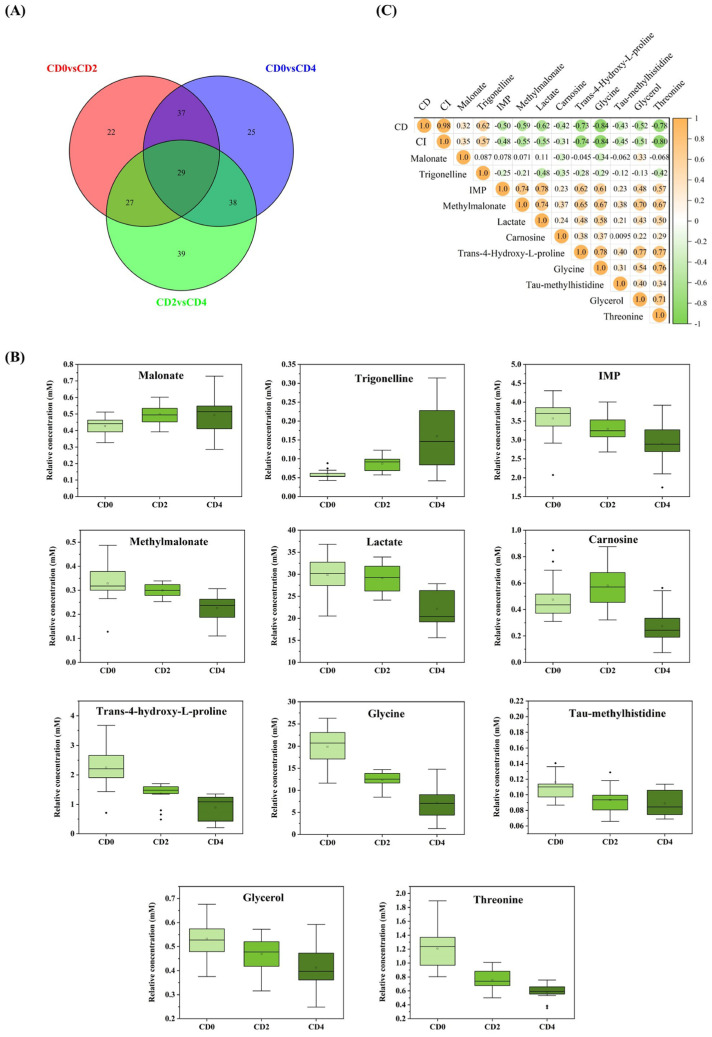
Differential metabolite analysis across crispness grades. (**A**) Venn diagram showing the number of unique and shared metabolites identified; (**B**) Box plots illustrating the relative concentration changes of key differential metabolites across crispness grades (CD0, CD2, CD4); (**C**) Heatmap of Pearson correlation coefficients between differential metabolites and both crispness grade (CD0, CD2, CD4) and composite crispness index (CI) scores.

The VIP score, derived from OPLS-DA, was used to quantify the degree of metabolite contribution to crispness classification. FC was used to measure relative differences in metabolite levels between comparison groups. Compounds corresponding to spectral regions with a VIP score > 1.0, FC > 2.0, and *p*-value < 0.05 were considered the most promising differential metabolites. A total of 115, 129, and 133 differential spectral regions were identified between the CD0 vs. CD2, CD0 vs. CD4, and CD2 vs. CD4 groups, respectively. A Venn diagram (Figure 4A) was used to analyze the screened differential spectral regions, revealing 29 common spectral regions across all three pairwise comparisons. A total of 11 compounds were identified from these 29 shared spectral regions and designated as differential metabolites, which account for the metabolic differences among groups with different crispness grades (Table 4). Changes in these metabolites effectively reflect the physiological alterations in tilapia as crispness increases. These metabolites include malonate, trigonelline, inosine monophosphate (IMP), methylmalonate, lactate, carnosine, trans-4-hydroxy-L-proline, glycine, τ-methylhistidine, glycerol, and threonine. A box plot (Figure 4B) was used to visualize the contents of these 11 differential metabolites in tilapia muscle across different crispness grades. Among them, the contents of trigonelline and malonate increased with increasing crispness; however, the increase in malonate was not statistically significant (*p* > 0.05). Compared with the CD0 group, the contents of the remaining nine metabolites were significantly decreased (*p* < 0.05). These results indicate that the crispy diet had a significant impact on the metabolism of tilapia.

A correlation analysis was conducted to evaluate the relationship between the 11 identified differential metabolites and the crispness of tilapia. The Pearson correlation coefficient (r) was used to quantify the degree of linear correlation between two variables; the strength of the correlation increases as the absolute value of r increases [28]. Figure 4C illustrates the correlations between all differential metabolites and both the crispness grade and composite index (CI) scores of crispy tilapia. Glycine, threonine, and trans-4-hydroxy-L-proline exhibited the strongest correlations with crispness, with r values of −0.84, −0.78, and −0.73, respectively. These metabolites can be considered as potential crispness markers.

### 3.5. Key Metabolic Pathway Analysis

**Figure 5:**
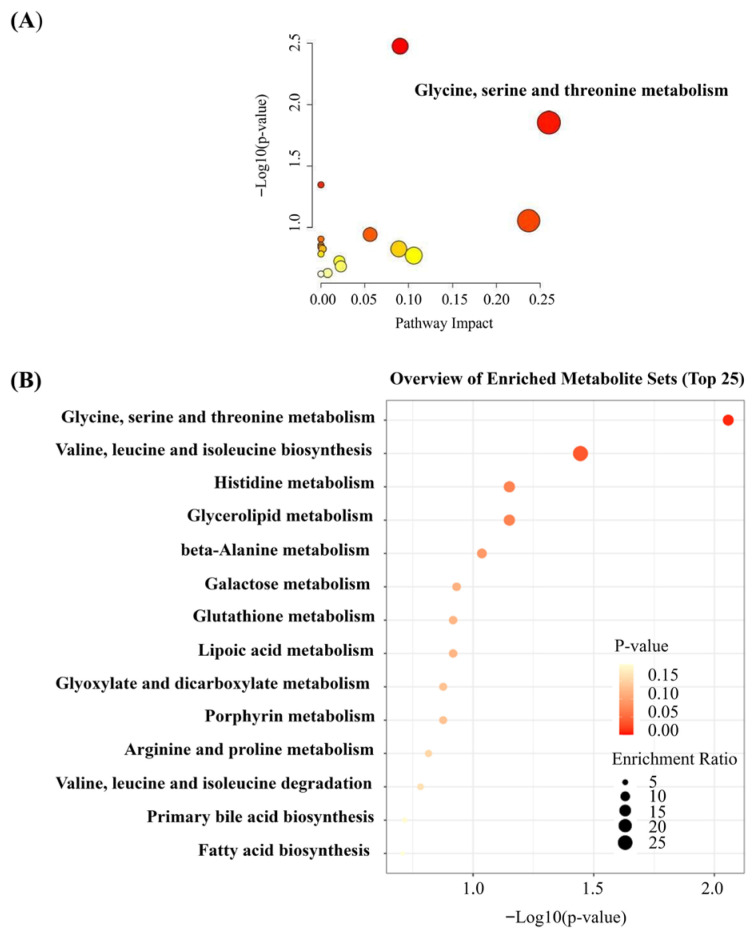
Pathway analysis of differential metabolites associated with tilapia crispness. (**A**) Metabolic pathway impact analysis showing significantly enriched pathways (*p* < 0.05); (**B**) Enrichment analysis overview displaying the most significantly enriched metabolic pathways. Note: (**A**) The colour of each dot represents the number of metabolites involved, and the size of the dot reflects the proportion of the pathway in the overall metabolic profile. The larger and darker the dot, the greater the impact of the pathway on overall metabolism. The Impact value on the horizontal axis represents the pathway impact value derived from pathway topology analysis. (**B**) The size of the bubble indicates the number of metabolites involved in the pathway. The colour of the bubble represents statistical significance: a darker colour corresponds to a lower *p*-value, indicating more significant changes in the involved metabolites or pathways.

To elucidate the potential mechanisms underlying the production of differential metabolites, the 11 identified metabolites were subjected to enrichment analysis and metabolic pathway analysis. Figure 5 shows that these 11 differential metabolites were enriched in 14 metabolic pathways. Among these pathways, three were significantly enriched in terms of biological functions (*p* < 0.05), namely “glycine, serine and threonine metabolism”, “valine, leucine and isoleucine biosynthesis”, and “histidine metabolism”. Pathway Impact reflects the importance of metabolic pathways; generally, pathways with an Impact value exceeding 0.1 are considered significant. The enrichment ratio reflects the number of differential metabolites involved in each pathway. In summary, based on the *p*-values, enrichment ratios, and Impact values presented in Figure 5, one major metabolic pathway was identified: glycine, serine, and threonine metabolism.

### 3.6. Histological Analysis of Muscle

**Figure 6:**
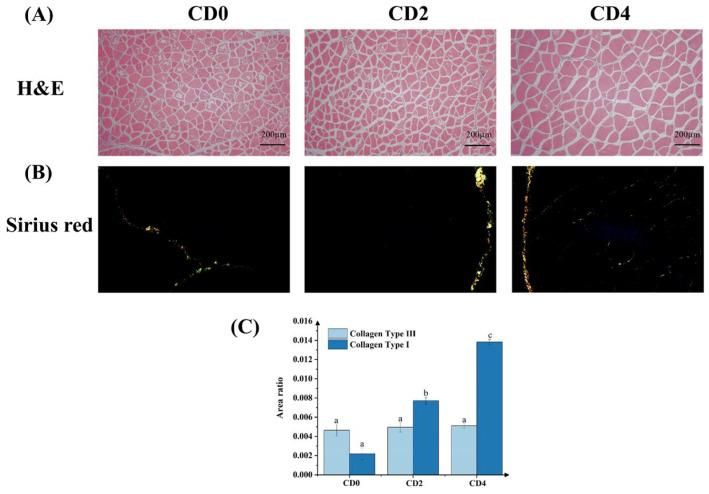
Histological analysis of tilapia muscle tissue with different crispness grades (CD0, CD2, CD4). (**A**) Representative images of muscle cross-sections stained with Hematoxylin and Eosin (H&E); (**B**) Representative images of muscle cross-sections stained with Sirius Red and visualized under polarized light (type I collagen appears as red-orange birefringence; type III collagen appears as green birefringence); (**C**) Relative quantification of type I and type III collagen percentages relative to total tissue area, calculated from Sirius Red-stained sections. Note: No significant difference was observed among groups marked with the same letter (*p* > 0.05).

Figure 6A illustrates the H&E-stained dorsal muscle tissue sections of tilapia. The analysis reveals that the myofibers predominantly exhibit irregular polygonal shapes, with distinct endomysium and perimysium visible across all three groups. No significant differences in myofiber diameter were observed between the CD2 and CD0 groups. However, the myofiber diameter was increased in the CD4 group. In Figure 6B, type I collagen appears yellowish-red, while type III collagen appears green. Although the content of type III collagen remained unchanged with increasing crispness grade, the content of type I collagen increased significantly (*p* < 0.05) (Figure 6C). Collagen was detected in both the endomysium and perimysium of the CD4 group. Notably, type I collagen was observed to surround type III collagen in the CD4 group.

## 4. Discussion

### 4.1. Effect of Crispy Flesh Culture on the Texture Properties of Tilapia

In this study, the CD4 group exhibited significantly higher hardness, chewiness, and gumminess than the CD0 group, with more pronounced enhancements in cooked fish fillets. Specifically, gumminess reflects the “tender yet elastic” sensory trait of crispy tilapia after prolonged cooking, whereas chewiness corresponds to its crispy texture. However, tilapia fed a standard control diet showed no significant changes in hardness, chewiness, elasticity, or other texture properties over 30, 60, and 90 days of feeding [11]. This suggests that dietary faba bean inclusion was the main factor driving fish texture, consistent with previous reports on grass carp and Yellow River carp [29,30].

Fish sensory properties are closely associated with their microstructural characteristics [31]. Skeletal muscle—the main edible part and quality determinant of fish flesh—consists of muscle fibres and intramuscular connective tissue (IMCT) [32]. Therefore, muscle fibres and connective tissue play a crucial role in shaping muscle texture. Muscle growth occurs via myofiber hyperplasia (increased number) and hypertrophy (increased diameter) [33]. Muscle hardness is negatively correlated with myofiber diameter but positively correlated with myofiber density [34]. In this study, myofiber diameter increased with crispness grade. This may be related to the growth stage of crispy tilapia, but it also suggests that muscle fibres are not the primary driver of increased crispness.

IMCT mainly comprises collagen and elastin fibres [35]. Meat texture is determined by collagen type, content, and distribution, with type I and type III collagen predominating in fish [36]. Increased type I collagen enhances fish texture hardness, whereas increased type III collagen improves elasticity. More collagen was observed in the perimysium of the CD4 group, contributing to firmer and more structured muscle tissue. Consequently, dietary faba bean supplementation enhances their crispness, potentially attributable to increased type I collagen content and collagen accumulation in the endomysium, both of which improve muscle hardness.

### 4.2. Effects of Crisp Flesh Culture on the Metabolism of Tilapia

#### 4.2.1. Crispy Flesh Culture Promotes Collagen Synthesis and Improves Muscle Texture

Our results showed significant differences in metabolites of crispy tilapia among the three crispness grades. In the CD4 group, muscle concentrations of certain amino acids (glycine, threonine, trans-4-hydroxy-L-proline, τ-methylhistidine) were decreased, suggesting enhanced amino acid metabolism with increasing crispness. Glycine, threonine, and trans-4-hydroxy-L-proline exhibited the most significant decreases. KEGG pathway analysis identified glycine, serine, and threonine metabolism as a key metabolic pathway. These changes are likely attributable to altered amino acid metabolism and protein synthesis. The modulation of the glycine-serine-threonine metabolic pathway observed in this study has also been reported in other NMR-based metabolomic investigations of tilapia under different environmental conditions [37].

Glycine, a non-essential amino acid, is primarily involved in protein synthesis, particularly collagen and elastin [38]. Glycine residues in collagen polypeptide chains are essential for stabilizing the triple-helical structure. Textural parameters (hardness, springiness, and chewiness) are strongly positively correlated with muscle collagen content [39]. In addition, glycine participates in creatine synthesis, which enhances the connective tissue network surrounding myofibers by providing more creatine, thereby improving muscle texture [40]. Thus, glycine may improve tilapia crispness by promoting collagen synthesis.

Threonine can increase glycine production by enhancing threonine dehydratase (TDH) activity [41]. Sirius red staining further confirmed glycine’s effect on collagen synthesis: compared with ordinary tilapia (CD0), type I collagen content in crispy tilapia (CD4) increased more than fivefold. Type I collagen was deposited both around muscle bundles and in the endomysium in the CD4 group. This indicates that glycine and threonine in crispy tilapia enhance specific muscle texture by promoting type I collagen synthesis.

Muscle firmness is associated with greater collagen stability [36]. Trans-4-hydroxy-L-proline is essential for forming intramolecular hydrogen bonds, which maintain collagen fibril integrity and contribute to the thermal stability of the triple-helical structure [42]. The lower trans-4-hydroxy-L-proline content in the CD4 group further indicates enhanced collagen synthesis, consistent with glycine content changes and Sirius red staining results. Due to its structural similarity to protein glycosylation sites, carnosine protects collagen by effectively inhibiting protein glycosylation through self-sacrifice [43]. Collagen, rich in glycosylation sites, is prone to binding free sugars, potentially disrupting its native spatial structure. Lower trans-4-hydroxy-L-proline and carnosine contents were detected in the CD4 group, suggesting that crispness changes relate not only to collagen quantity and type but also to its stability.

τ-Methylhistidine is a component of skeletal muscle contractile proteins. During muscle contraction, it is released via myofibrillar proteolysis [44]. Decreased τ-methylhistidine content in crispy tilapia suggests that crisp-enhancing culture may reduce myofibrillar proteolysis, thereby contributing to increased muscle mass.

Faba bean-containing diets accelerate amino acid metabolism, promote type I collagen synthesis and stabilization, and increase endomysial collagen deposition. This structural enhancement likely accounts for the greater hardness, gumminess, and chewiness observed in crispy tilapia.

#### 4.2.2. Crispy Flesh Culture Accelerates Energy Metabolism

Metabolic profiling showed that crispy tilapia (CD2 and CD4) exhibited lower levels of lactate and methylmalonate than ordinary tilapia (CD0). As a major circulating carbohydrate fuel, lactate is produced by muscle glycolysis under hypoxic conditions [45]. Recently, lactate has been widely recognized as an important energy substrate and signalling molecule. Methylmalonate enters the tricarboxylic acid cycle (TAC) via succinyl-CoA [46]. Reduced lactate and methylmalonate levels may be attributed to accelerated tilapia muscle growth under crisp-enhancing culture, which increases the demand for non-glycolytic precursors to meet the energy requirements for growth and development. This is consistent with previous NMR-based metabolomic studies in fish, in which changes in energy metabolism intermediates have been linked to altered growth rate and muscle quality [47]

#### 4.2.3. Crispy Flesh Culture Facilitates Tilapia Muscle Cell Growth

Crispy tilapia (CD2 and CD4) fed a faba bean diet exhibited lower levels of inosine monophosphate (IMP) compared to ordinary tilapia (CD0). IMP is a common umami compound in fish, indicating that crisp-enhancing culture reduces flavour intensity. Asaduzzaman et al. reported that dietary IMP supplementation promoted muscle cell growth by upregulating the mRNA expression of growth-related genes [48]. In the present study, crispy tilapia had larger myofiber diameters, which were negatively correlated with IMP content.

#### 4.2.4. Crispy Flesh Culture Accelerates Glycerol Metabolism

Glycerol and malonate are both involved in fatty acid metabolism. Fats are broken down into fatty acids and glycerol, and glycerol can subsequently be used for fatty acid synthesis. Malonate plays a critical role in the synthesis of malonyl-CoA, which is utilized by fatty acid synthases for the production of long-chain fatty acids and the chain elongation of existing fatty acids [49,50]. The higher malonate content and lower glycerol content observed in crispy tilapia suggest that faba bean-containing diets may inhibit lipolysis or accelerate glycerol metabolism. Peng et al. reported that the inclusion of faba bean meal in tilapia diets reduced muscle crude lipid content [6]. Therefore, faba beans may accelerate glycerol metabolism to provide energy for muscle growth. A negative correlation has been observed between muscle fat content and textural properties [51]. Collagen deposition rather than fat accumulation in muscle fibres is the primary reason for the enhanced crispness observed in the CD4 group. Both malonate and trigonelline levels were increased in crispy tilapia, likely due to their high abundance in legumes. The CD4 and CD2 groups were fed faba bean-containing crispy diets for a longer period, leading to higher accumulation of malonate and trigonelline in their bodies.

The systematic metabolic shift observed in our study is independently corroborated by a recent multi-omics investigation on the long-term effects of faba bean feeding in Nile tilapia. A 180-day regimen with a 60% faba bean-based diet led to a coordinated metabolic reprogramming characterized by activated lipid synthesis but inhibited amino acid and energy metabolism [52]. This aligns closely with our findings of reduced levels of amino acids (glycine, threonine) and energy intermediates (lactate), along with indicators of enhanced lipid and collagen deposition.

The limitations of this study should be acknowledged. First, the relatively small sample size may limit the generalizability of the findings, as the variability of characterized components could not be fully captured. Therefore, further validation of the 11 screened differential metabolites, including the three potential biomarkers, should be conducted with larger sample sizes, employing absolute quantification and more precise analytical instruments. Second, while this study focuses on the dynamic changes during the industrial crisping process, the lack of time-matched control groups fed a standard diet at 60 and 120 days makes it difficult to fully disentangle diet-induced effects from potential age-related metabolic changes. Future studies including such controls would help isolate the metabolic alterations attributable to dietary intervention. Nevertheless, the discovery of legume-specific biomarkers such as trigonelline, along with the specific modulation of the glycine-serine-threonine metabolic pathway—which is essential for collagen synthesis—strongly supports the primary role of the faba bean diet in promoting tilapia muscle crispness.

The specific modulation of the glycine-serine-threonine pathway merits further investigation to elucidate the molecular mechanisms by which faba bean components regulate collagen synthesis and muscle texture formation. Transcriptomic and proteomic analyses could complement the metabolomic findings to provide an integrated multi-omics perspective.

## 5. Conclusions

In summary, a ^1^H-NMR-based metabolomic approach was employed to systematically characterize the metabolite profiles of crispy tilapia across three distinct crispness grades (CD0, CD2, CD4). The findings explicitly demonstrate that muscle metabolic patterns in crispy tilapia vary significantly with the degree of crispness.

Furthermore, 11 differential metabolites were identified as key modulators regulating energy, amino acid, and lipid metabolic pathways. Specifically, these metabolites facilitate type I collagen synthesis, enhance collagen fibril structural stability, and accelerate triglyceride catabolic hydrolysis.

These metabolic perturbations are predominantly manifested as altered texture properties, fluctuations in myofibril diameter, and a marked elevation in type I collagen content. Collectively, these results provide critical mechanistic insights into the crispness formation during tilapia crisp-enhancing culture, and the identified differential metabolites may serve as novel molecular markers for tilapia crispness evaluation and targeted regulation.

## Figures and Tables

**Table 1:** Texture profile analysis (TPA) results of raw and cooked tilapia fillets with different crispness grades (CD0, CD2, and CD4).

Group		Hardness (g)	Springiness	Cohesiveness	Chewiness (g)	Resilience	Gumminess (g)
raw	CD 0	937.59 ± 334.80 ^b^	0.82 ± 0.11 ^a^	0.68 ± 0.03 ^b^	539.70 ± 241.44 ^b^	0.52 ± 0.05 ^b^	631.24 ± 213.14 ^b^
	CD 2	1192.75 ± 460.88 ^b^	0.71 ± 0.03 ^b^	0.74 ± 0.02 ^a^	624.50 ± 243.85 ^b^	0.62 ± 0.04 ^a^	879.40 ± 345.03 ^b^
	CD 4	1978.75 ± 395.54 ^a^	0.70 ± 0.04 ^b^	0.71 ± 0.02 ^a^	995.82 ± 231.93 ^a^	0.61 ± 0.05 ^a^	1415.26 ± 296.13 ^a^
cooked	CD 0	364.72 ± 31.93 ^b^	0.68 ± 0.05 ^b^	0.47 ± 0.03 ^b^	117.91 ± 16.82 ^b^	0.18 ± 0.01 ^c^	172.30 ± 20.22 ^b^
	CD 2	623.42 ± 219.16 ^ab^	0.86 ± 0.06 ^a^	0.73 ± 0.04 ^a^	395.02 ± 142.07 ^a^	0.40 ± 0.03 ^a^	457.67 ± 161.21 ^a^
	CD 4	914.43 ± 440.70 ^a^	0.80 ± 0.02 ^a^	0.72 ± 0.02 ^a^	523.74 ± 243.92 ^a^	0.37 ± 0.02 ^b^	655.32 ± 310.65 ^a^

Note: Values with different letters in the same line indicate significant differences (*p* < 0.05).

**Table 3:** Summary of identified metabolites in tilapia muscle tissue detected by ^1^H-NMR spectroscopy.

No.	Metabolites	^1^H Chemical Shift (ppm) ^a^	Assignments ^b^
1	Cholate	0.73 (s)	CH_3_
2	Pantothenate	0.92 (s)	CH_3_
3	Leucine	0.97 (t)	δCH_3_
4	Valine	1.00 (d); 1.05 (d)	γCH_3_; γ’CH_3_
5	Isoleucine	1.02 (d)	γCH_3_
6	Methylmalonate	1.23 (d)	βCH
7	Lactate	1.34 (d); 4.12 (q)	βCH_3_; αCH
8	Alanine	1.49 (d)	βCH_3_
9	Acetate	1.93 (s)	CH_3_
10	N_6_-Acetyllysine	1.99 (s); 3.18 (t)	CH_3_; NH_2_CH
11	Methionine	2.14 (s)	S-CH_3_
12	trans-4-Hydroxy-L-proline	2.17 (m);2.44 (m)	C_2_H_2_; C_2_′H_2_
13	Hydroxyacetone	2.17 (s)	CH_3_
14	3-Hydroxyisovalerate	1.27 (s); 2.38 (s)	αCH_2;_ βCH_3_
15	Glutamate	2.34 (m)	γCH_2_
16	Pyruvate	2.38 (s)	CH_3_
17	Succinate	2.41 (s)	CH_2_
18	Glutamine	2.45 (m); 3.78 (t)	γCH_2_; αCH
19	Dimethylamine	2.73 (s)	CH_3_
20	Sarcosine	2.75 (s)	CH_3_
21	N,N-Dimethylformamide	3.02 (s)	CH_3_
22	Trimethylamine	2.89 (s)	CH_3_
23	N-Methylhydantoin	2.92 (s)	CH_3_
24	N,N-Dimethylglycine	2.94 (s); 3.72 (s)	NCH_3_; CH_2_
25	Creatine	3.04 (s); 3.94 (s)	NCH_3_; CH_2_
26	Creatinine	3.06 (s); 4.05 (s)	NCH_3_; CH_2_
27	Malonate	3.14 (s)	CH_2_
28	Dimethyl sulfone	3.16 (s)	SCH_3_
29	Choline	3.21 (s)	NCH_3_
30	Phosphocholine	3.24 (s)	CH_3_
31	Glycerophosphocholine	3.25 (s)	CH_3_
32	Trimethylamine N-Oxide	3.27 (s)	CH_3_
33	Taurine	3.27 (t); 3.43 (t)	SCH_2_; NCH_2_
34	Glycine	3.57 (s)	CH_2_
35	Glycerol	3.66 (m)	CH_2_
36	Threonine	1.33 (d); 3.60 (d); 4.26 (m)	γCH_3_; αCH; βCH;
37	Arginine	3.78 (t)	CO-CH
38	Betaine	3.27 (s); 3.91 (s)	CH_3,_ CH_2_
39	Inosine monophosphate	4.02 (m); 4.05 (m); 4.38 (m); 4.53 (m); 6.16 (d); 8.24 (s); 8.58 (s)	C_5_′H_2_; C_4_′H; C_3_′H; C_2_′H; C_1_′H; C_1_H; C_4_H;
40	Trigonelline	4.44 (s); 9.13 (s)	NCH_3_; C_2_H
41	α-Glucose	5.24 (d); 3.54 (m); 3.90 (dd)	C_1_H; C_2_H; C_6_H_2_
42	β-Glucose	4.65 (d); 3.50 (m); 3.73 (dd)	C_1_H; C_2_H; C_6_H_2_
43	Inosine	6.11 (d); 8.24 (s); 8.35 (s)	C_1_′H; C_4_H; C_1_H;
44	Fumarate	6.53 (s)	CH
45	Carnosine	7.13 (s); 7.96 (s)	NCHC; NCHN
46	Phenylalanine	7.33 (m); 7.38 (m); 7.43 (m)	C_6_H; C_4_H; C_5_H
47	Niacinamide	8.72 (dd); 8.95 (d)	C_3_H; C_2_H
48	Xanthine	7.95 (s)	CH
49	Adenine	8.20 (s); 8.22 (s)	C_4_H; C_5_H
50	Tau-methylhistidine	3.69 (s); 7.03 (s); 7.73 (s)	NCH_3_; NCHC; NCHN
51	Pi-methylhistidine	3.68 (s); 7.94 (s)	NCH_3_; NCHN

Note: ^a^ Multiplicity of peaks: s, single peak; d, double peak; t, triple peak; q, quadruple peak; dd, double double peak; m, multiple peak; ^b^ Assignment: Reference to the HMDB (https://hmdb.ca) and PubChem databases (https://pubchem.ncbi.nlm.nih.gov).

**Table 4:** Relative concentrations of differential metabolites in tilapia muscle tissue across crispness grades (CD0, CD2, and CD4).

Metabolites	Binning (ppm)	Trend	Relative Concentration ( mM )		
			CD0	CD2	CD4
malonate	3.15		0.427 ± 0.014 ^a^	0.497 ± 0.014 ^a^	0.494 ± 0.029 ^a^
Trigonelline	4.27	↑	0.057 ± 0.003 ^b^	0.087 ± 0.005 ^b^	0.160 ± 0.023 ^a^
IMP	4.39	↓	3.568 ± 0.135 ^a^	3.293 ± 0.085 ^ab^	2.899 ± 0.137 ^b^
Methylmalonate	1.22	↓	0.328 ± 0.019 ^a^	0.299 ± 0.007 ^a^	0.226 ± 0.014 ^b^
Lactate	4.11	↓	29.854 ± 1.022 ^a^	29.114 ± 0.762 ^a^	22.115 ± 1.095 ^b^
Carnosine	7.13	↓	0.475 ± 0.040 ^a^	0.581 ± 0.041 ^a^	0.273 ± 0.035 ^b^
trans-4-hydroxy-L-proline	2.18	↓	2.249 ± 0.178 ^a^	1.357 ± 0.093 ^b^	0.892 ± 0.108 ^b^
Glycine	3.57	↓	19.860 ± 1.082 ^a^	12.418 ± 0.460 ^b^	7.152 ± 0.937 ^c^
Tau-methylhistidine	3.69	↓	0.116 ± 0.009 ^a^	0.093 ± 0.004 ^b^	0.089 ± 0.004 ^b^
Glycerol	3.67	↓	0.533 ± 0.023 ^a^	0.469 ± 0.017 ^ab^	0.413 ± 0.022 ^b^
Threonine	4.27	↓	1.208 ± 0.074 ^a^	0.756 ± 0.035 ^b^	0.584 ± 0.030 ^b^

Note: Values with different letters in the same line indicate significant differences (*p* < 0.05). ↑ indicates increased levels with crisping grade; ↓ indicates decreased levels with crisping grade.

## Data Availability

The original contributions presented in this study are included in the article and supplementary materials. Further inquiries can be directed to the corresponding authors.

## Supplementary Materials

The following supporting information can be downloaded at https://www.mdpi.com/article/10.3390/foods15071232/s1. Table S1: Detailed formulations of the two experimental diets; Table S2: Crispness evaluation criteria.

## Abbreviations

The following abbreviations are used in this manuscript:

^1^H-NMR	Proton nuclear magnetic resonance spectroscopy
PCA	Principal Components Analysis
OPLS-DA	Orthogonal Partial Least Squares–Discriminant Analysis
VIP	Variable Important in Projection
FC	Fold Change
EWM	Entropy weight method
TSP	2,2,3,3-Tetramethylsilane propionate
CI	Composite Index

## Appendix A. The Entropy Weight Method (EWM)

1. Analysis and Construction of a Comprehensive Index via EWM

EWM was employed to calculate the weight of each index based on the interrelationships among them. The composite index (CI) was constructed using the weights and normalized data. It should be noted that negative values must not appear during the calculation process. The specific calculation formulas and steps are as follows:

(1) Establishment of the raw index value matrix $A=\;{\left(a_{ij}\right)}_{m\times n}$:

(A1) $$\begin{array}{c} A\;=\;\left(\begin{array}{ccc} a_{11} & \ldots & a_{1n}\\ \vdots & \ddots & \vdots \\ a_{m1} & \ldots & a_{mn} \end{array}\right) \end{array}$$

where m is the number of samples and n is the number of indexes. *a_ij_* represents the corresponding TPA data.

(2) Normalization of matrix ***A*** to obtain matrix ***R***:

(A2) $$\begin{array}{c} R\;=\;\left(\begin{array}{ccc} r_{11} & \ldots & r_{1n}\\ \vdots & \ddots & \vdots \\ r_{m1} & \ldots & r_{mn} \end{array}\right) \end{array}$$

(A3) $$\begin{array}{c} r_{ij}=\frac{{a_{ij}-a}_{minj}}{\;a_{maxj}-a_{minj}} \end{array}$$

where ***R*** is the normalized matrix, *a_minj_* and *a_maxj_* are the minimum and maximum values in the j-th index, and $r_{ij}\in \left[0,\;1\right]$.

(3) Definition of entropy *h_j_*:

(A4) $$\begin{array}{c} h_{j}\;=\;-\frac{1}{lnm}\sum_{i\;=\;1}^{m}P_{ij}lnP_{ij} \end{array}$$

(A5) $$\begin{array}{c} P_{ij}=\frac{r_{ij}}{\sum_{i=1}^{m}r_{ij}}\; \end{array}$$

When $P_{ij}\;=\;0$, $P_{ij}lnP_{ij}\;=\;0$.

(4) Definition of entropy weight *w_j_*.

(A6) $$\begin{array}{c} w_{j}=\frac{1-h_{j}}{n-\sum_{j=1}^{n}h_{j}} \end{array}$$

$$w_{j}\in \left(0\right.,\left.1\right],\;\sum_{i=1}^{m}w_{j}=1$$

(5) Calculation of the composite index CI.

The CI was derived by weighting each normalized index with its corresponding entropy weight, as shown in the formula below:

(A7) $$\begin{array}{c} CI\;=\;\sum_{i\;=\;1}^{m}\left(r_{ij}w_{j}\right) \end{array}$$
