# Supplementary material for: Exploring Metabolite Changes in Crispy Tilapia During the Crisping Process via 1H-NMR Metabolomic Analysis
Source: Foods. 2026 Apr 4;15(7):1232. doi: 10.3390/foods15071232 (PMC13073416; doi:10.3390/foods15071232)
Supplement: Supplementary file 1 [file foods-15-01232-s001.zip › foods-4174217-supplementary.pdf]

Table S1. Detailed formulations of the two experimental diets.

Component	Regular Diet (%)	Crisp Diet (%)
Crude Protein	≥32.0	≥28.0
Crude Fiber	≤9.0	≤10.0
Crude Ash	≤10.0	≤15.0
Crude Fat	≥3.0	≥4.0
Total Phosphorus	≥0.7	≥1.0
Lysine	≥1.60	≥1.45
Moisture	≤12.5	≤12.5

Table S2. Crispness evaluation criteria.

Characteristics	Crispness graded
Excellent crispness, full elasticity, distinct chewiness, and the fish meat is not easily broken	4
Moderate crispness, good elasticity, certain chewiness, and the fish meat is not easily broken	3
Weak crispness, moderate elasticity, moderate chewiness, and the fish meat is easily broken	2
No crispness, poor elasticity, poor chewiness, and the fish meat is extremely easily broken	1 or 0
